# ANOSPEX: A Stochastic, Spatially Explicit Model for Studying *Anopheles* Metapopulation Dynamics

**DOI:** 10.1371/journal.pone.0068040

**Published:** 2013-07-08

**Authors:** Olugbenga O. Oluwagbemi, Christen M. Fornadel, Ezekiel F. Adebiyi, Douglas E. Norris, Jason L. Rasgon

**Affiliations:** 1 The Department of Entomology, Center for Infectious Disease Dynamics and Huck Institutes of the Life Sciences, Pennsylvania State University, University Park, Pennsylvania, United States of America; 2 W. Harry Feinstone Department of Molecular Microbiology and Immunology and the Johns Hopkins Malaria Research Institute, Johns Hopkins Bloomberg School of Public Health, Baltimore, Maryland, United States of America; 3 Department of Computer and Information Sciences, College of Science and Technology, School of Natural and Applied Sciences, Covenant University, Ota, Ogun State, Nigeria; Pennsylvania State University, United States of America

## Abstract

*Anopheles* mosquitoes transmit malaria, a major public health problem among many African countries. One of the most effective methods to control malaria is by controlling the *Anopheles* mosquito vectors that transmit the parasites. Mathematical models have both predictive and explorative utility to investigate the pros and cons of different malaria control strategies. We have developed a C++ based, stochastic spatially explicit model (ANOSPEX; ***Ano*** *pheles***Sp**atially-**Ex**plicit) to simulate *Anopheles* metapopulation dynamics. The model is biologically rich, parameterized by field data, and driven by field-collected weather data from Macha, Zambia. To preliminarily validate ANOSPEX, simulation results were compared to field mosquito collection data from Macha; simulated and observed dynamics were similar. The ANOSPEX model will be useful in a predictive and exploratory manner to develop, evaluate and implement traditional and novel strategies to control malaria, and for understanding the environmental forces driving *Anopheles* population dynamics.

## Introduction

Human malaria is one of the most important public health problems in many African countries, associated with high rates of mortality and morbidity. The disease presents with a spectrum of systemic complications ranging from mild and self-limiting illness to life-threatening pathology. Malaria incidence has increased in many areas of the African continent due to climate change, insecticide and drug resistance, and social/economic issues [1]–[4].

As an infectious disease, malaria is transmitted through the bite of infected female *Anopheles* mosquitoes. Thus, one of the most effective methods to control the disease is by controlling the mosquito vectors. Despite concerted efforts by governmental agencies, public and private non-governmental researchers and other relevant health agencies to offer effective control strategies, malaria still persists in many endemic regions of the world. Thus, there is an urgent need for the development and implementation of existing and novel malaria vector control interventions. Mathematical models are a crucial part of developing and optimizing control techniques, since they are one of the only ways to optimize deployment and conduct risk-assessment prior to an actual intervention attempt [5]–[7].

Mathematical modeling is crucial to understanding *Anopheles* population and transmission dynamics for developing strategies for disease control [8]. For over 100 years, models have been developed and applied towards the control of malaria, mosquitoes and mosquito-borne related diseases, ranging from simple models of vectorial capacity to complex predictive models of malaria epidemiology [9]–[17].

The most detailed individual-based models of mosquito populations have been developed for *Aedes aegypti*. Focks and colleagues developed the Container Inhabiting Mosquito Simulation Model (CIMSiM), a deterministic simulation model that is driven by empirical weather data and incorporates very detailed aspects of mosquito biology [18]–[19]. Although CIMSiM has shown utility in predicting mosquito dynamics in nature [20], it has several limitations: (1) it ignores stocasticity in the data, (2) it assumes a single panmictic mosquito population, and (3) it is written in VisualBasic which is not easily compilable on newer computers [21]. To address these issues, Magori and colleagues developed the SkeeterBuster model. SkeeterBuster shares many algorithms with CIMSiM (and in fact can recapitulate it exactly) but it is written C++, is stochastic and spatially explicit [21]. SkeeterBuster operates at the scale of individual water-filled containers for immature stages and individual properties (houses) for adults. SkeeterBuster also incorporates mosquito genetics [21].

Multiple simulation models of *Anopheles* population dynamics have been developed [22]–[28]. However, these models have their limitations. For instance, the *Anopheles* model developed by Depinay and colleagues [23] does not explicitly incorporate mating between male and female *Anopheles gambiae* mosquitoes and was not validated against field data. The models developed by Eckhoff [27] and White et al [28] did not present spatially explicit simulations. Arifin and colleagues [29] developed a detailed spatial agent-based model to show the influence of resources on mosquito populations. However, this model was not driven by empirical weather data, nor was it validated against field observations.

To address some of these issues, we developed a stochastic, spatially explicit model of *Anopheles* metapopulation dynamics. We call this model ANOSPEX, for “***Ano*** *pheles* **Sp**atially-**Ex**plicit”. ANOSPEX is biologically rich, driven by empirical weather data, and parameterized by field data to simulate *Anopheles* metapopulation dynamics. Simulation results from ANOSPEX were preliminarily validated post-hoc using empirical *Anopheles* adult female collection data from Macha, Zambia.

## Methods

### ANOSPEX Overview

ANOSPEX was written in C++ and Visual C++ programming languages on an Intel Pentium i^5^ Computing system running Windows 7. The ANOSPEX codes are a combination of new codes and codes implemented from Skeeterbuster. Parameters used in ANOSPEX were derived from literature whenever possible (Table S1). We modeled stochasticity in the same way as SkeeterBuster [18]; ANOSPEX is not deterministic. Also like SkeeterBuster, ANOSPEX is weather-driven. Hourly weather data (2009–2011) were obtained from the Malaria Institute at Macha (MIAM), Zambia. Weather parameters used in the model were maximum temperature, minimum temperature, average temperature, precipitation, saturation deficit and relative humidity. ANOSPEX does not include mosquito genetics, only mosquito metapopulation dynamics. ANOSPEX is implemented as a grid representation of residential properties, where each property has one house and two larval habitats for mosquitoes to develop in. Adult mosquitoes can move from one property to another property as described below. For simulations, breeding habitats were initially seeded with 25 eggs. References for parameter values are available as supplementary material.

### Model Development and Metapopulation Dynamics of Immature and Adult *Anopheles* Mosquitoes

**Figure 1:**
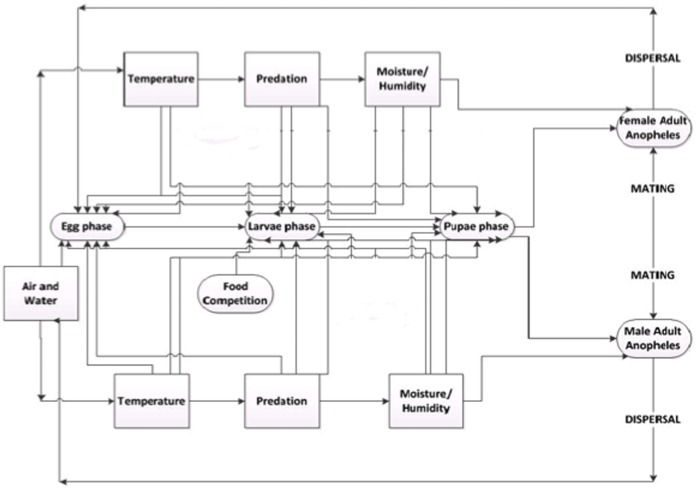
General ANOSPEX model flowchart.

The diagram in Figure 1 shows the schematic container model of *Anopheles* lifecycle and development for ANOSPEX.

### Egg Phase

An important aspect of the African *Anopheles* ecology is the egg phase [21]. Important factors that affect the hatching and survival of *Anopheles* eggs include predation, water and air temperatures, sun exposure, and water depth of the breeding containers [21], [24], [30]–[32]. The prominent role temperature plays in *Anopheles* egg development cannot be over-emphasized [31], [37]–[41]. All malaria vectors are poikilothermic in nature [33]. In ANOSPEX, we applied the enzyme kinetics model derived by Sharpe and DeMichele [34], which is based on the absolute rate of reaction of enzymes for temperature-dependent developmental rates [18], [21], [36], [42]–[43].

In ANOSPEX, for *Anopheles* eggs to successfully hatch, the average water temperature has to be above 21°C and eggs have to be consistently immersed in water [31]. If the water within the container is below the average hatching temperature or the eggs are not immersed they will not hatch [44]. If eggs are mature and immersed, eggs hatch [35] according to the enzyme kinetics model (eq. 1) [18], [21], [34], [36]. The fundamental assumption is that a single control enzyme regulates poikilothermic development and the reaction rate of this enzyme affects and determines the rate of development of the organism (here, *Anopheles*) [34], [36], [45], [46].
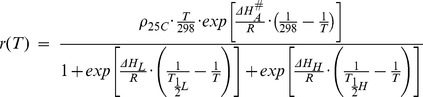
(1)


*r(T)* is the rate of development per hour at temperature *T(°K)*_._ 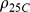
is the developmental rate per hour at 25**°**C. 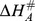
represents the enthalpy of activation of the reaction catalyzed by the enzyme (cal·mol-1); 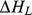
is the low temperature inactivation enthalpy change associated with the enzyme (cal·mol-1); 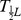
represents the temperature in °K where 50% of the enzyme is inactivated by low temperature. 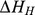
is the high temperature inactivation enthalpy change associated with the enzyme (cal·mol-1); 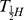 is the temperature in °K where 50% of the enzyme is inactivated by high temperature. R is the universal gas constant, with a value of (1.987cal·mol-1) [18], [21], [34], [36], [47]. The set of parameter values obtained from [21] was applied to the egg phase modeling within ANOSPEX: 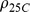 = 0.0413; 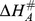 = 1.0000; 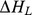 = −170644; 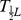 = 288.8; 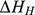 = 1000000; and 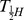 = 313.3.

The egg hatch algorithm within sites is shown in Figure S1.

### Larval Phase

The development rate and survival of *Anopheles* larvae is dependent on water temperature [48], implying that the *Anopheles* larvae phase also depends on the enzyme kinetic equation (eq. 1) for development [21]. The set of parameter values obtained from [21] was applied to the larval phase modeling within ANOSPEX; 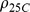 = 0.037; 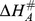 = 15684; 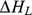 = −229902; 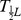 = 286.4; 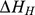 = 822285; and 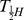 = 310.3. Other factors within an African locality context, such as land cover types and topography [49], habitat types [49]–[53], predators [53]–[57], food availability [58], competition [59]–[60] and desiccation [61]–[65] also affect the development, survival and distribution of *Anopheles* larvae within their habitats.

In ANOSPEX, there are two conditions that *Anopheles* larvae must meet before pupating. First, larvae must attain complete physiological maturity. In ANOSPEX, larvae attain physiological maturity if their cumulative development exceeds a threshold value (eq. 2). The second condition is that developed *Anopheles* larvae attain pupation only if they have attained a sufficient weight worthy of them pupating. *Anopheles* larvae undergo a developmental cycle based on the enzyme kinetic equation as illustrated in eq.1 until they attain 4^th^ instar [18], [21].

The algorithm governing the development of *Anopheles* larvae within containers is depicted in the flowchart in Figure S2. For a given cohort of age n at time t, the cumulative physiological development CD_t_ is given by equation 2 [15], [16], [18]:
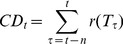
(2)


In ANOSPEX, we assumed that the probability of larval development is a function of the total physiological development. Thus, no larvae matured below a total physiological development of 0.92 and above 1.20 respectively [18]. Setting these conditions allows certain portions of the *Anopheles* larval cohort to achieve maturity at a lower cumulative development, while rest achieve higher than the mean date of physiological maturation before being developed.

In ANOSPEX, food intake by *Anopheles* larvae contributes to the increase in individual and collective larval weight. Food intake was based on an average of 3-day food intake plus random food intake by the *Anopheles* larvae [66]–[67]. The dynamics of the amount of *Anopheles* larval food in a breeding site and the larvae cohort weight are governed by equations adapted from CIMSiM [15], [68].

Daily survival rates for *Anopheles* eggs, larvae, pupae and adults were determined and estimated from the literature (Table S1). Larvae that die are converted into biomass as larval food. We estimated the value of this parameter with a 0.40 conversion factor [58], [69].

### Pupation Phase

*Anopheles* pupation was modeled as in CIMSiM and SkeeterBuster [15]–[16], [18]. Developing *Anopheles* larvae have to achieve a specific weight at maturation to successfully transit into the pupae phase. Temperature and the cumulative physiological development of *Anopheles* larvae are two factors that affect the transit into the pupae phase. The set of parameter values obtained from [21] was applied to the pupae phase modeling within ANOSPEX; the values of 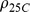 = 0.034; 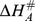 = 1.0000; 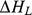 = −154394; 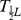 = 313.8; 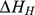 = 554707; and 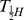 = 313.8.

The model flowchart for the pupae phase is shown in Figure S3. Completion of the *Anopheles* pupae developmental phase occurs as soon as *Anopheles* pupae attain complete maturation. We assumed that the maturation probability for an *Anopheles* pupa was a function of its total physiological development. We assumed that no pupa attains maturity below a total physiological development value of 0.92 while all pupae above 1.20 attain maturity. The survival of *Anopheles* pupae in ANOSPEX model depends on temperature. Dead *Anopheles* pupae are converted into biomass for food, with a conversion rate of 0.40 [58], [69].

### Adult Phase

The emergence of adult *Anopheles* from their pupal case leads into the adult phase of the *Anopheles* life cycle. *Anopheles* pupae that successfully enter into this phase further develop into male and female *Anopheles* adults. Both male and female *Anopheles* adult mosquitoes can undergo mortality due to extreme conditions from the local environment.

Female adult *Anopheles* mosquitoes’ gonotrophic development was also modeled based on the enzyme kinetics equation outlined above [18], [21], [34], [36]. The set of parameter values obtained from [21] was applied to the female adult gonotrophic phase modeling within ANOSPEX; the values of 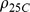 = 0.02; 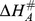 = 1000; 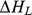 = −75371; 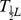 = 293.1; 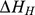 = 388691; and 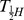 = 313.4.

The algorithm governing the development of *Anopheles* male and female adult development within containers is depicted in the flowcharts of Figures S4 and S5 respectively. We assumed unrestricted access of female adult *Anopheles* to blood, and the availability and homogeneity of hosts. Female *Anopheles* adults were assumed to oviposit after the completion of their gonotrophic cycle.

### Adult Mosquito Movement

*Anopheles* mosquito movements were modeled by adapting knowledge gained from cellular automata, by using the Von Neumann neighborhood algorithm [70]–[72], where for each dispersing adult *Anopheles* mosquito there is a random selection of one of the possible four directions. Each residential property on the grid is represented by the coordinates 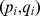. Distance between one residential property 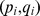 and the other 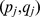 is represented by 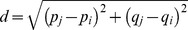. In ANOSPEX, we estimated that each adult *Anopheles* has a short-range dispersal probability of 0.35 [73]–[75]. We also applied the boundary assumptions adopted in the SkeeterBuster model [18].

### Field Survey and Preliminary Model Validation

We validated ANOSPEX by comparing predicted numbers of female *Anopheles* against mosquito capture data from Macha, Zambia. The choice of Macha, Zambia as a study location for our experiment was made because of the availability of hourly weather data from MIAM (Johns Hopkins Malaria Institute at Macha) that was coincident with previously published mosquito collection data (February–April 2009) (CDC traps, cattle-baited traps, and human landing captures [76].

## Results

### Mosquito Population Dynamics

**Figure 2:**
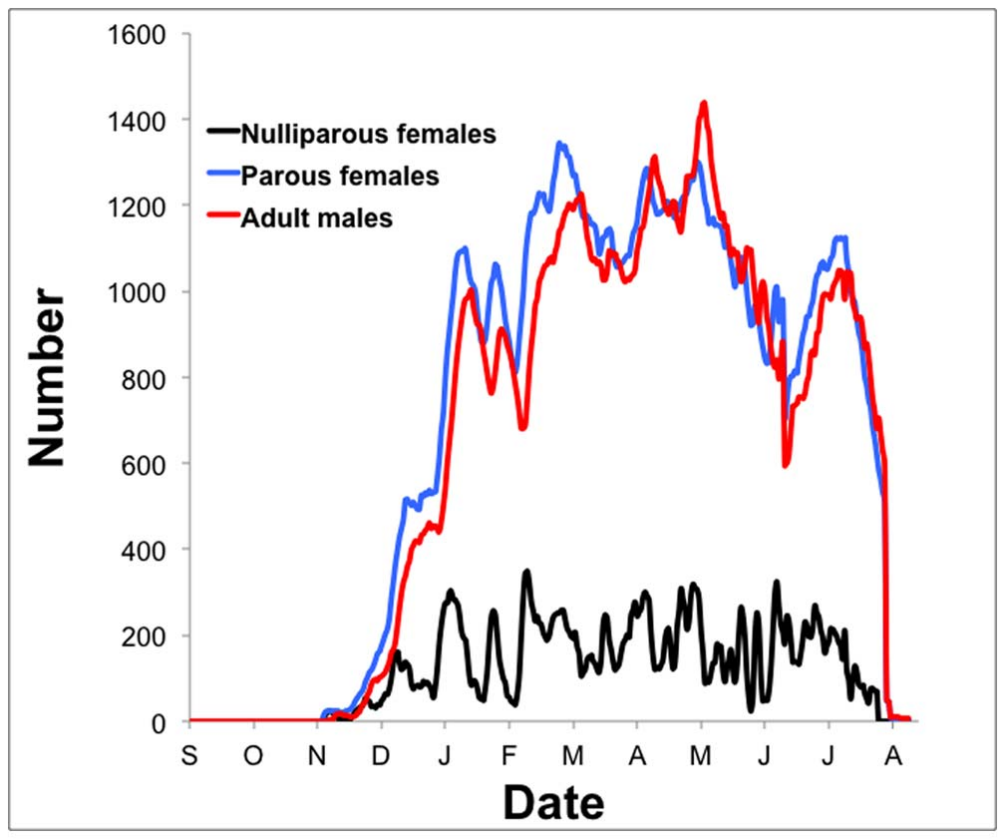
Simulation run results for *Anopheles* adult dynamics over a 10X10 grid. Letters represent the first letter of the months of the year.

The total number of adult mosquitoes (males, nulliparous females and parous females) over a 10×10 grid was simulated over a one-year period to evaluate the role of weather in governing mosquito population dynamics. ANOSPEX captures the weather-driven dynamics and shows, unsurprisingly, that mosquito population numbers peak during the rainy season, reach an approximate equilibrium level, then crash during the dry season (Figure 2).

### Mosquito Dispersal

**Figure 3:**
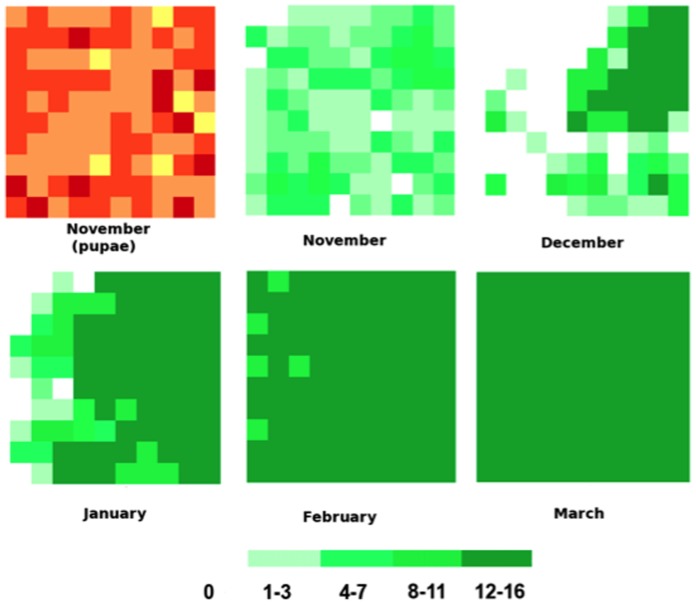
Simulation run results for *Anopheles* female adult dynamics over a 10×10 grid from the onset to the peak of the wet season. First box represents initial pupal distribution among properties.

ANOSPEX simulates mosquito population dynamics across a grid of residential properties. Mosquito numbers within a property are a product of local production (driven by weather) and dispersal of adults into and out of the property. Figure 3 shows an example of this dynamic for female adult *Anopheles* in a 100-property grid (10×10) from the onset to the peak of the wet season (November – March). Mosquito numbers within and between properties change due to reproduction, death and migration.

### Preliminary Model Validation

Model validation is critical to ascertaining the utility of a predictive model. In order to validate ANOSPEX, we compared model predictions from a 25-property grid (5×5) to empirical adult female *Anopheles* mosquito collection numbers from the Johns Hopkins Malaria Institute at Macha during the time period of February 1 to April 10, 2009 [76]. ANOSPEX was driven by empirical weather data for this same time period. Since ANOSPEX simulates adult numbers but does not distinguish particular habitats, we pooled data from CDC light traps, cattle-baited traps, and human landing catches (both inside and outside houses).

**Figure 4:**
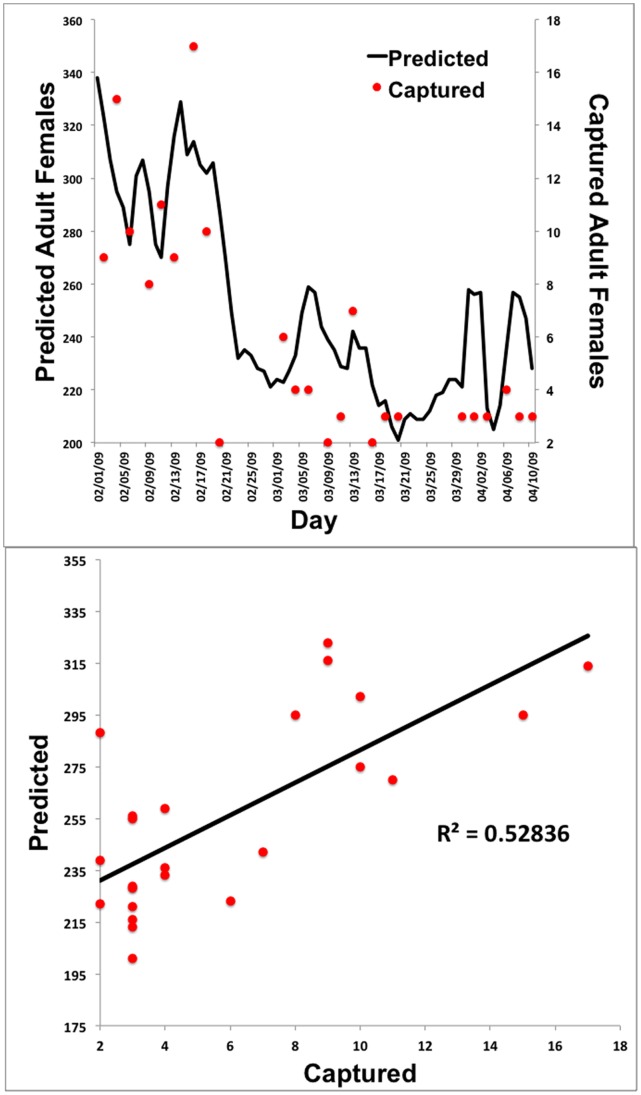
Preliminary ANOSPEX validation. A: Predicted numbers of female adult *Anopheles arabiensis* mosquitoes were compared to empirical mosquito collection data from Macha, Zambia [76]. B: Correlation between predicted and empirical results.

While the overall number of mosquitoes differed significantly between predicted and empirical results, the trends were similar (Figure 4A). This is to be expected, since ANOSPEX simulates total mosquito numbers, while the collection data represented that fraction of the mosquito population that was captured, and simulations were carried out over a 5×5 grid that may not accurately reflect the geographic size of the natural habitat. Nevertheless, ANOSPEX performed well at capturing the overall mosquito population dynamics, as both the predicted and empirical mosquito numbers change by approximately the same magnitude (Figure 4). The correlation between model predicted and empirical results was highly significant (P<0.0001), with an R^2^ of 0.53 (Figure 4B).

## Discussion

From an applied standpoint, many novel vector control strategies (such as release of genetically modified mosquitoes) cannot be empirically tested under true field conditions before an actual intervention attempt. Mathematical models are a crucial first step to assess control strategies for safety and efficacy prior to implementation. Models are also useful for improving the implementation of traditional control measures. From a basic standpoint, models are useful for investigating the environmental factors that govern mosquito population dynamics.

The most detailed models of mosquito population dynamics have been previously developed for *Aedes aegypti*. Similar models for *Anopheles* mosquitoes are needed, especially in light of recent interest in novel strategies for control of malaria. ANOSPEX is a flexible model that can be customized to fit any area of interest, by modifying the underlying property setup and weather files. As currently coded, ANOSPEX simulations will be exceeding useful for examining the control strategies based on population suppression, such as insecticide usage [77]–[81], RIDL (Release of Insects carrying a Dominant Lethal) [82]–[87] habitat modification [88]–[89], or lethal densovirus [90]–[92]. ANOSPEX will also be useful to examine the impact of environmental change on mosquito population dynamics. ANOSPEX currently lacks a mosquito genetics component, but this could easily be added to investigate population replacement strategies based on genetic modification or *Wolbachia* symbionts [93]–[95].

### Model Limitations

It has been said, “all models are wrong, but some are useful” [96]. ANOSPEX is no exception. ANOSPEX results are based on the complex interaction of many parameters, all with varying degrees of uncertainty. It is likely that we have overemphasized the impact of some parameters, while possibly missing others that are important. Sensitivity analysis of model parameters will help to refine model results. Nevertheless, our preliminary validation results indicate that ANOSPEX can provide a reasonable description of the dynamics of *Anopheles* populations. As ANOSPEX is further developed and refined, it will be a useful tool to understand *Anopheles* population dynamics and develop malaria control strategies.

## Supporting Information

Figure S1 Daily egg hatching flowchart for hatch probabilities within containers.
(PDF)

Figure S2 *Anopheles* larvae development flowchart.
(PDF)

Figure S3 Flowchart for emergence probabilities of *Anopheles* pupae within a container.
(PDF)

Figure S4 Flowchart for *Anopheles* male adult development.
(PDF)

Figure S5 Flowchart for *Anopheles* female adult development.
(PDF)

Table S1 References for parameter values used in simulations.
(XLS)
