# Supplementary figures and images for: ANOSPEX: A Stochastic, Spatially Explicit Model for Studying Anopheles Metapopulation Dynamics
Source: PLoS One. 2013 Jul 8;8(7):e68040. doi: 10.1371/journal.pone.0068040 (PMC3704604; doi:10.1371/journal.pone.0068040)

Fig S1

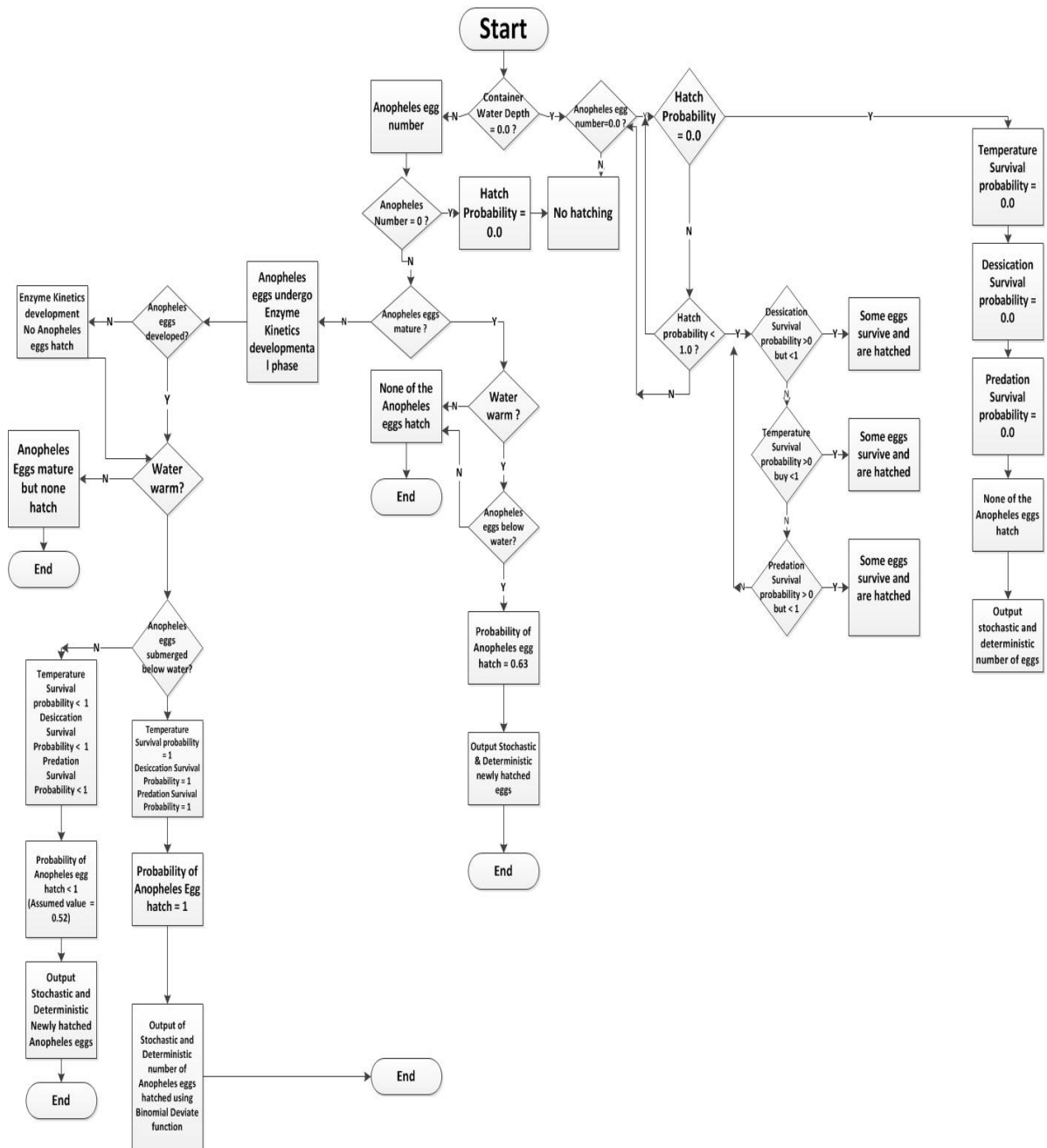

Supplement: Figure S1 — Daily egg hatching flowchart for hatch probabilities within containers. (PDF) [file pone.0068040.s001.pdf]

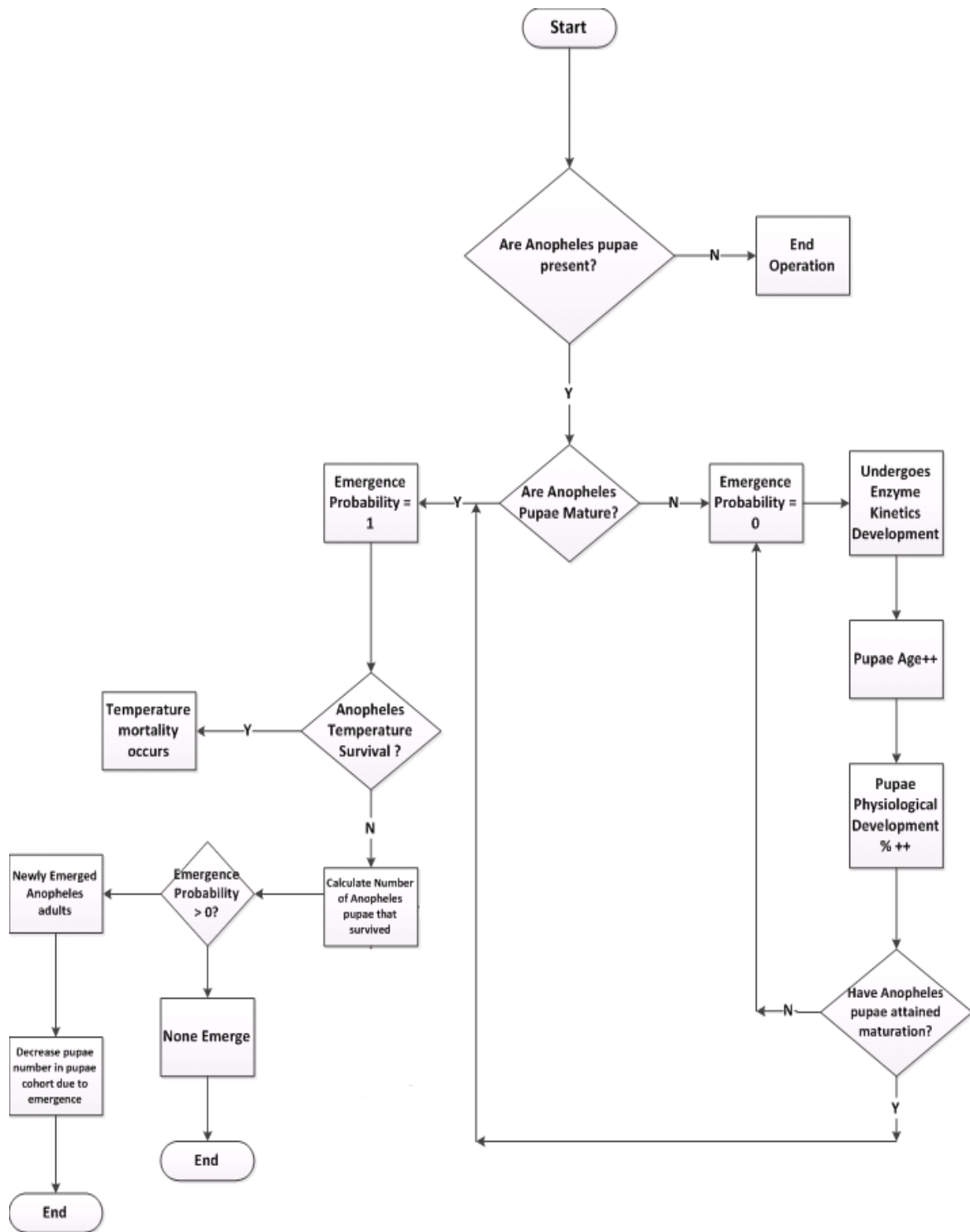

Fig S3

Supplement: Figure S3 — Flowchart for emergence probabilities of Anopheles pupae within a container. (PDF) [file pone.0068040.s003.pdf]

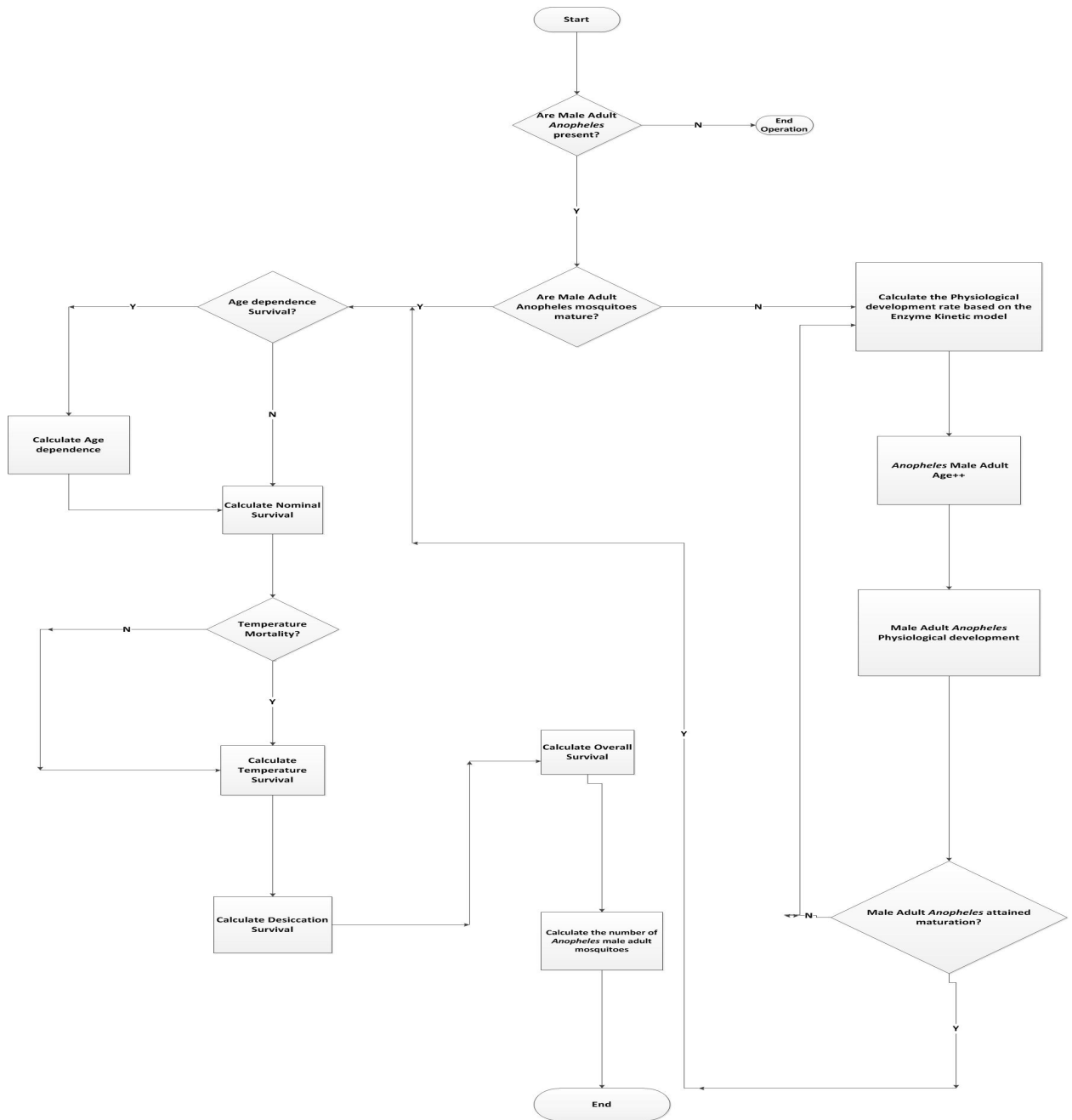

Fig S4

Supplement: Figure S4 — Flowchart for Anopheles male adult development. (PDF) [file pone.0068040.s004.pdf]
