# Supplementary material for: ANOSPEX: A Stochastic, Spatially Explicit Model for Studying Anopheles Metapopulation Dynamics
Source: PLoS One. 2013 Jul 8;8(7):e68040. doi: 10.1371/journal.pone.0068040 (PMC3704604; doi:10.1371/journal.pone.0068040)
Supplement: Figure S2 — Anopheles larvae development flowchart. (PDF) [file pone.0068040.s002.pdf]

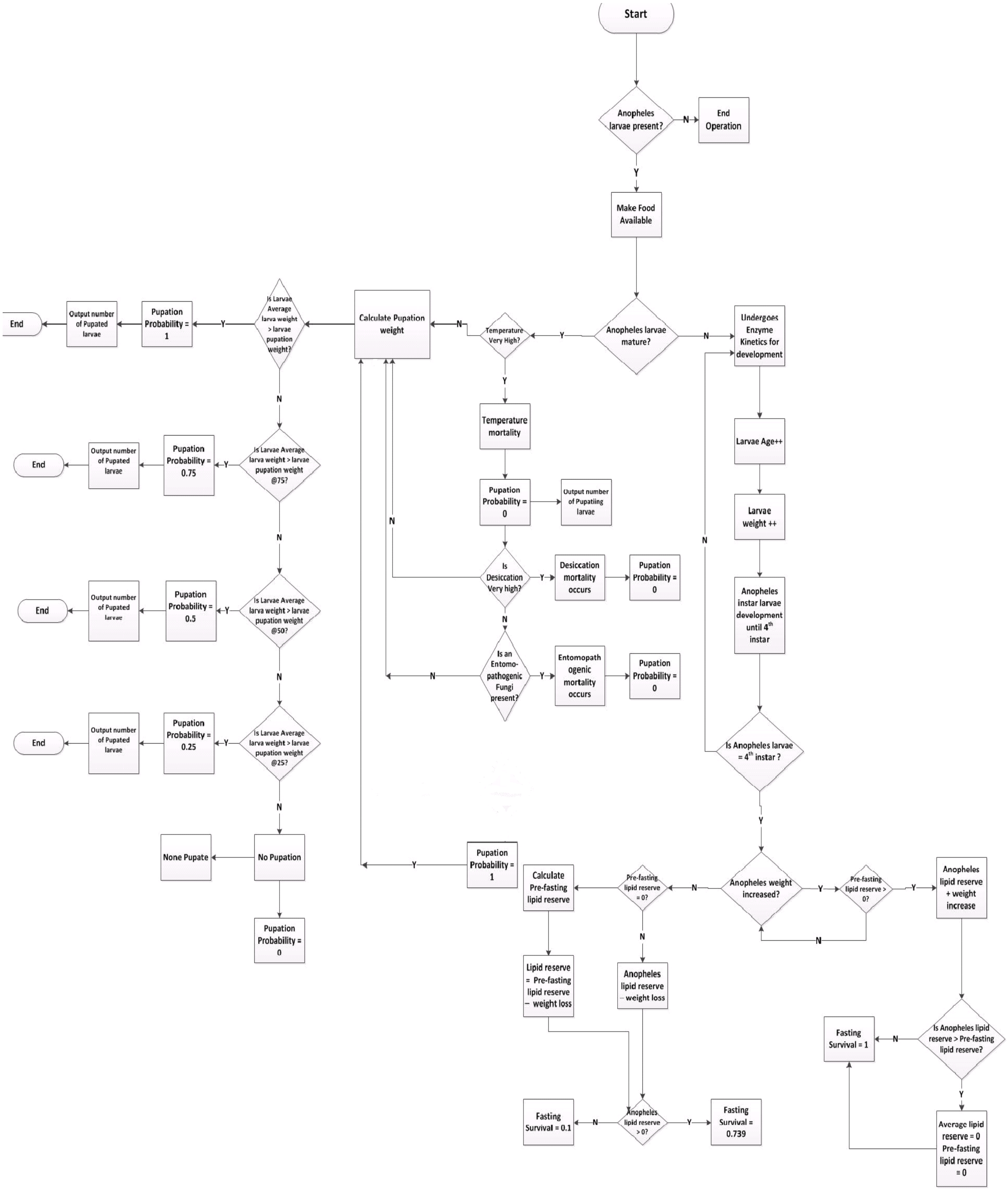

Start
Anopheles larvae present?
End Operation
Make Food Available
Anopheles larvae mature?
Undergoes Enzyme Kinetics for development
Larvae Age++
Larvae weight ++
Anopheles instar larvae development until 4th instar
Is Anopheles larvae = 4th instar ?
Temperature Very High?
Temperature mortality
Pupation Probability = 0
Output number of Pupating larvae
Is Desiccation Very high?
Desiccation mortality occurs
Pupation Probability = 0
Is an Entomo-pathogenic Fungi present?
Entomopath ogenic mortality occurs
Pupation Probability = 0
Calculate Pupation weight
Is Larvae Average larva weight > larvae pupation weight?
Pupation Probability = 1
Output number of Pupated larvae
End
Is Larvae Average larva weight > larvae pupation weight @75?
Pupation Probability = 0.75
Output number of Pupated larvae
End
Is Larvae Average larva weight > larvae pupation weight @50?
Pupation Probability = 0.5
Output number of Pupated larvae
End
Is Larvae Average larva weight > larvae pupation weight @25?
Pupation Probability = 0.25
Output number of Pupated larvae
End
No Pupation
None Pupate
Pupation Probability = 0
Pupation Probability = 1
Anopheles weight increased?
Pre-fasting lipid reserve > 0?
Anopheles lipid reserve + weight increase
Pre-fasting lipid reserve = 0?
Calculate Pre-fasting lipid reserve
Lipid reserve = Pre-fasting lipid reserve – weight loss
Anopheles lipid reserve – weight loss
Anopheles lipid reserve > 0?
Fasting Survival = 0.1
Fasting Survival = 0.739
Is Anopheles lipid reserve > Pre-fasting lipid reserve?
Fasting Survival = 1
Average lipid reserve = 0 Pre-fasting lipid reserve = 0
Y
N

Fig S2
