# Supplementary material for: ANOSPEX: A Stochastic, Spatially Explicit Model for Studying Anopheles Metapopulation Dynamics
Source: PLoS One. 2013 Jul 8;8(7):e68040. doi: 10.1371/journal.pone.0068040 (PMC3704604; doi:10.1371/journal.pone.0068040)
Supplement: Figure S5 — Flowchart for Anopheles female adult development. (PDF) [file pone.0068040.s005.pdf]

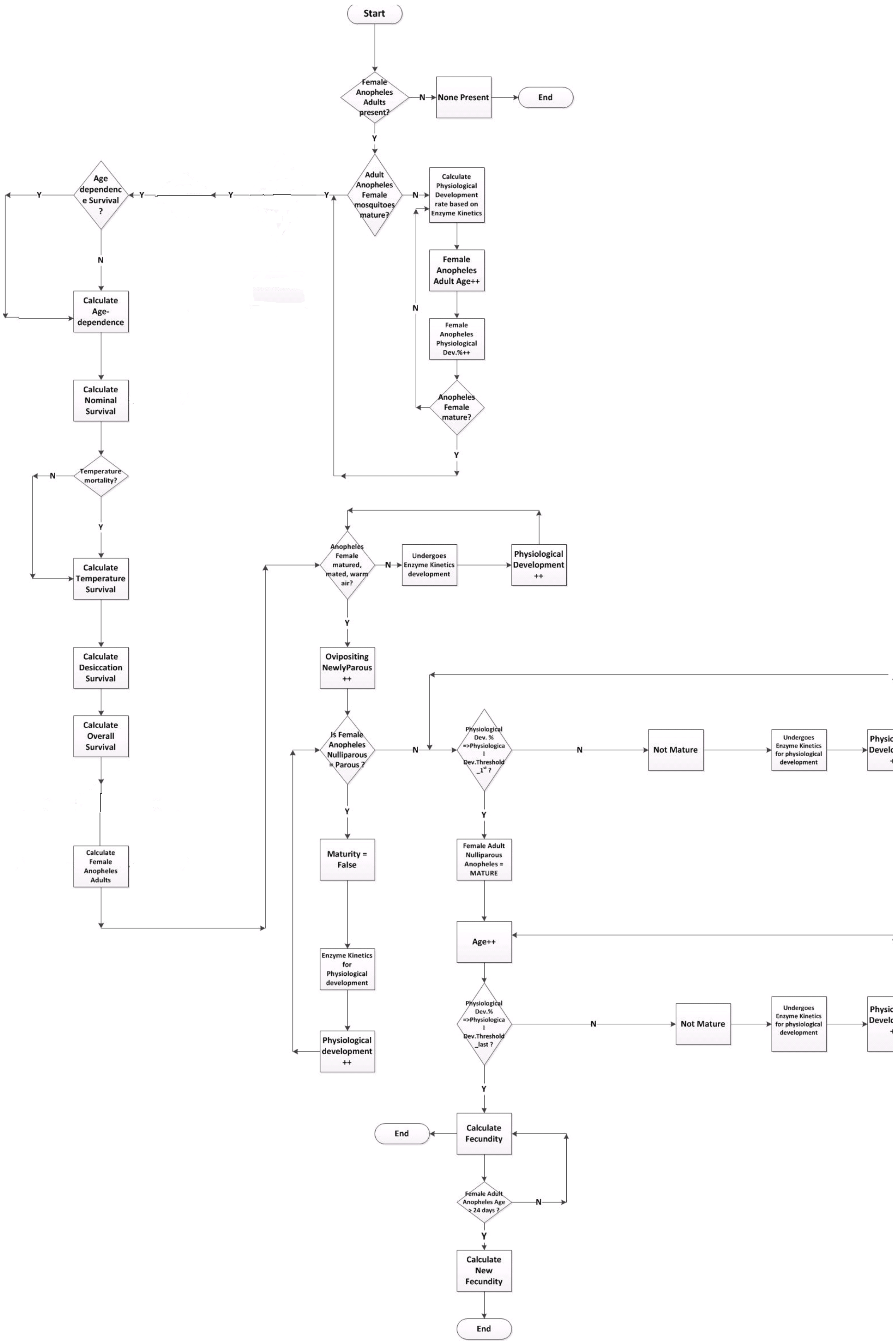
Start
Female Anopheles Adults present?
N
None Present
End
Y
Adult Anopheles Female mosquitoes mature?
Calculate Physiological Development rate based on Enzyme Kinetics
Female Anopheles Adult Age++
Female Anopheles Physiological Dev.%++
Anopheles Female mature?
Age dependence Survival ?
Calculate Age-dependence
Calculate Nominal Survival
Temperature mortality?
Calculate Temperature Survival
Calculate Desiccation Survival
Calculate Overall Survival
Calculate Female Anopheles Adults
Anopheles Female matured, mated, warm air?
Undergoes Enzyme Kinetics development
Physiological Development ++
Ovipositing NewlyParous ++
Is Female Anopheles Nulliparous = Parous ?
Maturity = False
Enzyme Kinetics for Physiological development
Physiological development ++
Not Mature
Undergoes Enzyme Kinetics for physiological development
Female Adult Nulliparous Anopheles = MATURE
Age++
Calculate Fecundity
Female Adult Anopheles Age > 24 days ?
Calculate New Fecundity

Fig S5
